# SERINC5: One antiviral factor to bind them all

**DOI:** 10.1371/journal.ppat.1011076

**Published:** 2023-01-19

**Authors:** Uddhav Timilsina, Spyridon Stavrou

**Affiliations:** Department of Microbiology and Immunology, Jacobs School of Medicine and Biomedical Sciences, University at Buffalo, New York, United States of America; University of Wisconsin-Madison, UNITED STATES

## SERINC5 biology and function

Serine Incorporator (SERINC) family members are multi-pass transmembrane proteins, whose putative role is the incorporation of serine into membranes during phosphatidylserine and sphingolipid biosynthesis [1]. SERINC proteins are highly conserved among eukaryotes with homologues identified in *Saccharomyces cerevisiae* [2]. Mammalian genomes encode 5 SERINC paralogs (SERINC1-5), which share substantial amino acid homology [1]. Of the 5 members of the SERINC protein family, SERINC5 is the most studied.

SERINC5 was initially identified in cultured rat oligodendrocytes [3] and mouse brain tissues [4], indicating a possible biological role in the central nervous system. In fact, a genome-wide association study revealed an association of human *SERINC5* SNPs with borderline personality disorder [5]. Nevertheless, human SERINC5 has low tissue specificity and is expressed in various tissues including placenta, skeletal muscle, spleen, thymus, testis, leukocytes, heart, and liver [6]. While human *SERINC5* encodes multiple splice isoforms, only the longest *SERINC5* transcript variant encodes a stable protein comprised of 10 transmembrane domains (TMs) [7]. Recent reports have shown that human and drosophila SERINC proteins have a bipartite organization, in which 2 subdomains are bisected by a long diagonal helix forming a lipid binding groove [8]. Although, the physiological role of SERINC5 is not fully understood, SERINC5 has recently emerged as an antiviral factor targeting a diverse number of viruses from unrelated families.

## SERINC5 as an antiviral factor

In 2015, 2 independent studies highlighted the antiviral function of SERINC proteins for the first time [9,10]. It was shown that human SERINC5, and to a lesser extent SERINC3, restrict human immunodeficiency virus-1 (HIV-1) and murine leukemia virus (MLV) infectivity in the absence of the retroviral accessory proteins Negative factor (Nef) and glycosylated Gag (glycoGag), respectively, by targeting virus–cell membrane fusion in the target cell [9,10]. Subsequent studies found that the antiretroviral activity of SERINC5 is evolutionarily conserved, as even *Drosophila melanogaster* SERINC, which shares only 36% amino acid sequence identity with human SERINC5, potently restricted HIV-1 infectivity [8]. Similarly, the murine homologue of SERINC5, mSERINC5, restricts MLV infection both in vivo and in vitro [11,12]. Additionally, in the past year, studies have reported that the antiviral effect of SERINC5 extends to other viral families.

We recently found that SERINC5 restricts Severe Acute Respiratory Syndrome Coronavirus 2 (SARS-CoV-2) infectivity in the absence of ORF7a, a SARS-CoV-2 accessory protein [13]. Recent studies reported that SERINC5 inhibits influenza A virus (IAV) infection as well, although, no viral protein was identified to counteract the antiviral effect of SERINC5 [14,15]. Furthermore, hepatitis B virus (HBV) and classical swine flu virus (CSFV) were found to be blocked by SERINC5 [16,17]. Pseudoviruses generated using envelope glycoproteins from feline endogenous retrovirus (RD144), rabies virus, and lymphocytic choriomeningitis virus (LCMV) were also reported to be sensitive to SERINC5 restriction [18], yet further research in the context of autologous virions is needed. Contrary to classical antiviral restriction factors, SERINC5 is not under strong positive selection [19]. Finally, *SERINC5* expression levels are not induced by HIV-1, MLV, or SARS-CoV-2 infection [9,11,13].

## SERINC5 mechanism of restriction

**Fig 1:**
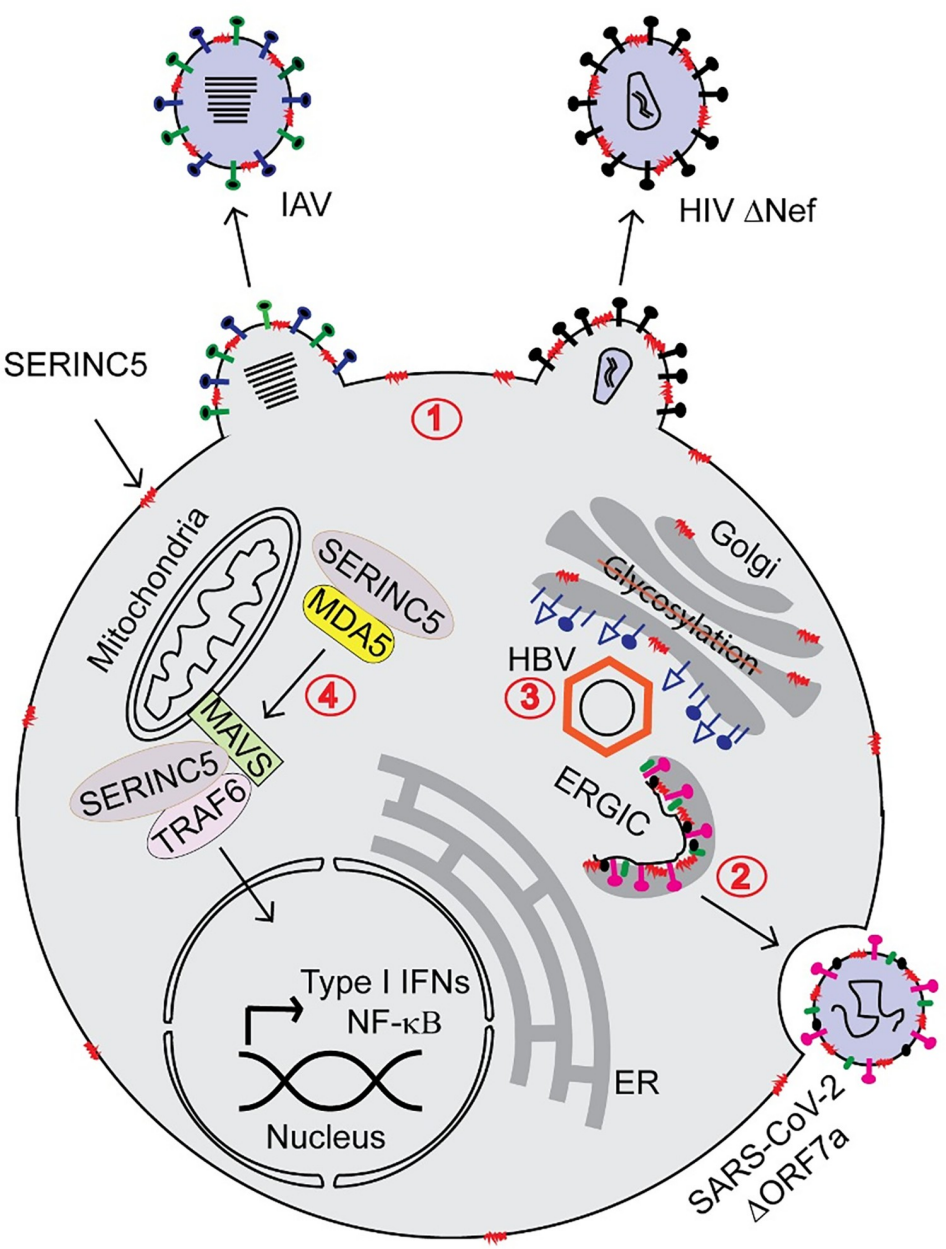
**Schematic of SERINC5 antiviral action:** SERINC5 is incorporated into the nascent virions at the plasma membrane, where IAV and retroviruses (HIV) assemble and bud (1) and at the ERGIC, the site for coronavirus assembly (2). SERINC5 interferes with the glycosylation of HBV surface proteins at the Golgi (3) and enhances the host innate immune response by interacting with nucleic acid sensor (MDA5) and adaptor proteins (MAVS, TRAF6) (4). ERGIC, endoplasmic reticulum–Golgi intermediate compartment; HBV, hepatitis B virus; HIV, human immunodeficiency virus; IAV, influenza A virus.

SERINC5 localizes to the plasma membrane lipid rafts (assembly sites for HIV-1 and IAV) [20] and the endoplasmic reticulum–Golgi intermediate compartment (ERGIC; assembly site for SARS-CoV-2) [13]. In the case of retroviruses, SARS-CoV-2 and IAV, incorporation of SERINC5 in budding virions is needed to block virus–cell membrane fusion (Fig 1) [9,11,13–15]. However, the exact mechanism of SERINC5-mediated inhibition of virion fusion is still not well understood. Multiple studies have suggested that SERINC5 targets the HIV-1 envelope glycoprotein (Env), thereby blocking virus–cell membrane fusion [21–23]. Studies involving single virion fluorescence microscopy revealed that virion-associated SERINC5 affects virus–cell fusion by changing the conformation or clustering of HIV-1 Env [21,22]. Another recent report found that SERINC5- containing HIV-1 particles were unable to complete the final fusion pore expansion step and remained as fusion intermediates [23]. Though SERINC5 is thought to affect lipid biosynthesis [1], virion-incorporated SERINC5 does not affect the lipid composition and organization of HIV-1 particles [24]. However, it was shown that local changes in viral membrane lipid order following treatment with lipophilic drugs like amphotericin B alleviated SERINC5 restriction on HIV-1 membrane fusion [23] indicating a possible role of SERINC5 interaction with lipids at the membrane interface. Finally, studies have shown that the route of virus entry (pH dependent versus independent) does not affect SERINC5-mediated restriction of virus entry [11,14,15,18]. It is worth noting that in the case of IAV, not only virion-associated SERINC5 but also SERINC5 expressed in target cells restricted virion entry, as SERINC5 present in the target cells also inhibited IAV HA-mediated fusion [14]. Interestingly, the mechanism utilized by SERINC5 to inhibit HBV is distinct from that of retroviruses, SARS-CoV-2, and IAV (Fig 1). SERINC5 interferes with the glycosylation of HBV surface proteins (HBs) resulting in decreased HBV virion release from the producer cells [16].

Recent reports have shown that SERINC5 can also modulate the type I IFN response (Fig 1). Different studies showed that SERINC5 can interact with melanoma differentiation-associated protein 5 (MDA5), as well as mitochondrial antiviral signaling protein (MAVS) and tumor necrosis factor receptor-associated factor (TRAF6), 2 adaptor proteins essential for antiviral innate immunity, to enhance type I IFN production and nuclear factor κB (NF-κB) signaling [17,25]. Moreover, proinflammatory cytokine production was found to be enhanced in monocyte-derived macrophages infected with HIV-1 containing SERINC5 [26]. Collectively, these studies suggest that SERINC5 can also modulate infection as an innate immunity factor.

## Viral antagonists of SERINC5

Viruses have evolved mechanisms to counteract the inhibitory effect of SERINC5. To date, HIV-1/SIV Nef, MLV glycoGag, EIAV S2, and SARS-CoV-2 ORF7a proteins have been shown to antagonize the antiviral effect SERINC5 [9,10,13,27]. Nef and S2 are associated with the plasma membrane via N-terminal myristoylation, whereas glycoGag and ORF7a are integral transmembrane proteins. All known viral antagonists of SERINC5 utilize a conserved strategy to counteract SERINC5; they reduce the surface levels of SERINC5 in producer cells, thereby blocking SERINC5 incorporation in nascent virions [9,10,13,27]. Nef, glycoGag, and S2 proteins interact with adaptor protein 2 (AP2) to facilitate internalization of SERINC5 from the plasma membrane to endosome/lysosome for degradation, thereby excluding its incorporation from the nascent virions [9,10,27]. Studies on the effect of Nef on SERINC5 found that Nef N-terminal region (residues 32 to 39) is crucial for its interaction with SERINC5, while the C-terminal endocytic sorting motif (E_160_xxxLL_165_) mediates binding with AP2 [9,28]. Furthermore, it has been shown that Cyclin K/Cyclin-dependent kinase 13 (CDK13) complex-mediated phosphorylation of a serine residue (S360) within the intracellular loop 4 (ICL4) of SERINC5 enhances Nef-AP2-SERINC5 interaction [29]. However, S360 residue is not conserved among SERINCs [8]. Moreover, the AP2-binding tyrosine-based sorting motif (Y_36_XXL_39_) and 2 additional residues P31 and R63 of glycoGag are critical for glycoGag-mediated down-regulation of SERINC5 [12]. In the case of ORF7a, the only known non-retroviral viral antagonist of SERINC5, its transmembrane domain is critical for its anti-SERINC5 activity [13]. Further studies identifying novel viral antagonists of SERINC5 are needed.

## Envelope as a determinant of SERINC5 resistance

Viral envelope (Env) glycoproteins impart differential sensitivity to SERINC5 restriction. HIV-1/MLV pseudoviruses containing envelope glycoproteins from other viruses such as vesicular stomatitis virus (VSV), Ebola, avian leukosis virus (ALV), ecotropic MLV, human T-cell lymphoma virus-1 (HTLV-1), or parainfluenza virus 5 (PIV5) are resistant to SERINC5 restriction [9–11,18,30]. These SERINC5-resistant glycoproteins do not affect SERINC5 incorporation into the virions, indicating that viral Envs intrinsically determine the sensitivity to SERINC5 activity. Amphotropic and xenotropic MLVs are highly sensitive to SERINC5 restriction, whereas ecotropic MLV is resistant [9,11,30]. Certain HIV-1 strains (HXB2, NL4-3, and 89.6) are also highly sensitive, whereas others (JFRL, AD8-1, and YU-2) are completely resistant to SERINC5 activity [31]. Nevertheless, SERINC5 in the virions sensitizes HIV-1 to neutralizing antibodies targeting the membrane-proximal external region (MPER) of the gp41 transmembrane subunit and the gp41-gp120 interface [8,31]. Recently, a structure-based mutagenesis screening identified that SERINC5 residues within the third and the fifth extracellular loops (ECL3 and ECL5) and in proximity to the interface between the subdomains are important for both HIV-1 restriction and sensitization of the MPER of HIV-1 Env to neutralizing antibodies [8]. In an effort to understand what aspects of the Env render it sensitive to SERINC5 restriction, a previous report identified the third hypervariable (V3) loop in the exterior subunit (gp120) of the HIV-1 Env as critical in determining whether the Env is resistant or sensitive to SERINC5 [31]. It is noteworthy that the V3 loop is the major determinant of HIV-1 cell tropism and contributes to HIV-1 Env trimer stability [32]. Moreover, the HIV-1 Env cytoplasmic tail was reported to affect resistance or sensitivity to SERINC5 restriction by modifying Env conformation [33]. Collectively, the observations reported so far indicate that HIV-1 Env conformation and stability determine its sensitivity to SERINC5. However, a recent study [18] indicated that SERINC5 sensitivity of certain envelope glycoproteins is influenced by viral core as well. Similar to HIV-1, IAV strains show differential sensitivity to SERINC5 restriction, which is determined by the N-linked glycosylation pattern of IAV HA [14]. Interestingly, in the case of SARS-CoV-2, Spike glycoproteins derived from different SARS-CoV-2 variants (Alpha, Beta, Gamma, and Delta) are all sensitive to SERINC5 restriction [13]. The influence of other viral Env glycosylation patterns on SERINC5 sensitivity needs to be further studied.

## Concluding remarks

In summary, SERINC5, while initially identified as an antiretroviral restriction factor, growing evidence suggests that it has a broad antiviral activity targeting viruses from a wide number of virus families. Interestingly, it is not under positive selection and is not inducible by IFN. Although multiple studies have been conducted investigating the antiviral effect of SERINC5, its cellular functions remain largely unknown. Recent findings showing that SERINC5 modulates viral infection as an innate immunity factor suggest that the role of SERINC5 is more complex than previously thought. Future studies integrating the different aspects of SERINC5 during virus infection are needed. Finally, a better understanding on the repertoire of viruses affected by SERINC5 and the factors viruses encode to counteract it will provide a more accurate understanding on the antiviral role of SERINC5.
